# Prediction of significant prostate cancer in biopsy-naïve men: Validation of a novel risk model combining MRI and clinical parameters and comparison to an ERSPC risk calculator and PI-RADS

**DOI:** 10.1371/journal.pone.0221350

**Published:** 2019-08-26

**Authors:** Jan Philipp Radtke, Francesco Giganti, Manuel Wiesenfarth, Armando Stabile, Jose Marenco, Clement Orczyk, Veeru Kasivisvanathan, Joanne Nyaboe Nyarangi-Dix, Viktoria Schütz, Svenja Dieffenbacher, Magdalena Görtz, Albrecht Stenzinger, Wilfried Roth, Alex Freeman, Shonit Punwani, David Bonekamp, Heinz-Peter Schlemmer, Markus Hohenfellner, Mark Emberton, Caroline M. Moore

**Affiliations:** 1 Department of Urology, University Hospital Heidelberg, Heidelberg, Germany; 2 Department of Radiology, German Cancer Research Center, Heidelberg, Germany; 3 Department of Radiology, University College London Hospital NHS Foundation Trust, London, United Kingdom; 4 Division of Surgery & Interventional Science, University College London, London, United Kingdom; 5 Division of Biostatistics, German Cancer Research Center, Heidelberg, Germany; 6 Department of Urology, University College London Hospitals NHS Foundation Trust, London, United Kingdom; 7 Division of Oncology/Unit of Urology, URI, IRCCS Ospedale San Raffaele, Milan, Italy; 8 Vita-Salute San Raffaele University, Milan, Italy; 9 Institute of Pathology, University of Heidelberg, Heidelberg Germany; 10 Institute of Pathology, University Medicine Mainz, Mainz, Germany; 11 Department of Pathology, University College Hospital, London, United Kingdom; 12 Centre for Medical Imaging, University College London Hospitals NHS Foundation Trust, University College London, London, United Kingdom; Medical University of Vienna, AUSTRIA

## Abstract

**Background:**

Risk models (RM) need external validation to assess their value beyond the setting in which they were developed. We validated a RM combining mpMRI and clinical parameters for the probability of harboring significant prostate cancer (sPC, Gleason Score ≥ 3+4) for biopsy-naïve men.

**Material and methods:**

The original RM was based on data of 670 biopsy-naïve men from Heidelberg University Hospital who underwent mpMRI with PI-RADS scoring prior to MRI/TRUS-fusion biopsy 2012–2015. Validity was tested by a consecutive cohort of biopsy-naïve men from Heidelberg (n = 160) and externally by a cohort of 133 men from University College London Hospital (UCLH). Assessment of validity was performed at fusion-biopsy by calibration plots, receiver operating characteristics curve and decision curve analyses. The RM`s performance was compared to ERSPC-RC3, ERSPC-RC3+PI-RADSv1.0 and PI-RADSv1.0 alone.

**Results:**

SPC was detected in 76 men (48%) at Heidelberg and 38 men (29%) at UCLH. The areas under the curve (AUC) were 0.86 for the RM in both cohorts. For ERSPC-RC3+PI-RADSv1.0 the AUC was 0.84 in Heidelberg and 0.82 at UCLH, for ERSPC-RC3 0.76 at Heidelberg and 0.77 at UCLH and for PI-RADSv1.0 0.79 in Heidelberg and 0.82 at UCLH. Calibration curves suggest that prevalence of sPC needs to be adjusted to local circumstances, as the RM overestimated the risk of harboring sPC in the UCLH cohort. After prevalence-adjustment with respect to the prevalence underlying ERSPC-RC3 to ensure a generalizable comparison, not only between the Heidelberg and die UCLH subgroup, the RM`s Net benefit was superior over the ERSPC`s and the mpMRI`s for threshold probabilities above 0.1 in both cohorts.

**Conclusions:**

The RM discriminated well between men with and without sPC at initial MRI-targeted biopsy but overestimated the sPC-risk at UCLH. Taking prevalence into account, the model demonstrated benefit compared with clinical risk calculators and PI-RADSv1.0 in making the decision to biopsy men at suspicion of PC. However, prevalence differences must be taken into account when using or validating the presented risk model.

## Introduction

The decision-making process of prostate cancer (PC) diagnosis is still controversial. The traditional approach we have taken in diagnosing PC, with raised prostate specific antigen and transrectal prostate ultrasound-guided (TRUS)-biopsy, do not appear to result in overall mortality benefit yet do lead to overdiagnosis and overtreatment of indolent disease [1]. The introduction of multiparametric magnetic resonance imaging (mpMRI) of the prostate has significantly improved the diagnostic accuracy of radiology in PC [2–7]. This has led to a higher detection rate of clinically significant PC (sPC) by means of targeted biopsies [7–9]. In addition, approaches using upfront MRI and targeted biopsies help to reduce the diagnosis of clinically indolent disease [7–9].

The implementation of clinical data with mpMRI findings has become of significant importance for urologists in order to better stratify those men who may deserve (or not) a prostate biopsy [10,11]. At this regard, there has been a considerable interest in developing new clinical tools and multivariable risk calculators (RC) and models (RM) able to predict the probability of a patient to harbour sPC, including data from mpMRI [10–15]. There is evidence suggesting that the addition of mpMRI findings to clinical information could increase the accuracy of diagnosis, but only few of these models have been either internally or externally validated [10,11,15]. Therefore, there is a need of further robust studies to develop more accurate RMs [16].

The purpose of this study was to validate a previously published RM predicting the probability of harbouring clinical sPC based on clinical parameters and mpMRI features focusing on cohorts of biopsy-naïve men undergoing mpMRI and subsequent prostate biopsy [10].

## Material and methods

### Study population

The study population comprised 160 consecutive biopsy-naïve patients from University Hospital Heidelberg and 139 men from University College London Hospital (UCLH). At Heidelberg, patients were enrolled and registered into a prospective database assessing MRI-targeted/TRUS-fusion biopsy between 2012 and 2017. Institutional review board approval was obtained (S011/2011) and all subjects provided written informed consent. Subgroups were previously reported [10,17]. The original RM-development contained data of patients undergoing MRI and fusion-biopsy between 2012–2015 [10]. Data of the 160 consecutive subjects in the present study, who underwent MRI and fusion-biopsy from 2016–2017, were not used in original RM-development. At UCLH, institutional review board exemption was granted from the local joint research office. The subgroup from UCLH consisted of 139 consecutive men undergoing transperineal prostate biopsy agreed to be sampled with additional targeted biopsy directed to suspicious lesions on prostate mpMRI from 2010–2012.

Data were retrospectively analysed. Inclusion criteria were suspicion of PC based on prostate specific antigen (PSA) or digital rectal examination (DRE), mpMRI with Prostate Imaging Reporting and Data System (PI-RADS) version 1.0 scoring according to the European Society of Urogenital Radiology guidelines and fusion-biopsy [18]. Men under active surveillance and men who had missing data were excluded (S1 Fig). Of all 299 biopsy-naive men, full data on PI-RADSv1.0, biopsy-outcome, PSA, age, DRE, prostate volume (PV), PSA-density, lesions on TRUS and the European Randomised Study of Screening for Prostate Cancer Risk calculator (ERSPC-RC) 3 were available for 293 men (160 from Heidelberg and 133 from UCLH). Those samples served for validation and for comparisons to ERPSC-RC3, PI-RADSv1.0 and combined ERSPC-RC3 and PI-RADSv1.0.

### Imaging

All mpMRI examinations were performed using a 3-Tesla-system (Magnetom, Siemens, Erlangen, Germany) using a multichannel-body-surface coil at Heidelberg and two scanners (one 1.5 Tesla- and one 3-Tesla-scanner (Avanto and Verio, Siemens, Erlangen, Germany) at UCLH, as previously reported [10,19]. In Heidelberg, all image analyses were prospectively performed according to PI-RADSv1.0 by or under supervision of expert uroradiologists (HPS, DB, MCR, 7–18 years of experience in prostate-MRI). In this scoring system every parameter T2WI, DWI and DCE is scored on a five-point scale separately. Additionally, each lesion is given an overall score, to predict its chance of being a clinically sPC. According to the PI-RADS guidelines for Version 1, no dominant sequence for different zones in which the lesion occur, as e.g. DWI for peripheral zone in Version 2, were applied.

For the UCLH cohort, each scan was retrospectively reported by a radiologist highly-experienced in prostate-MRI according to PI-RADSv1.0 (FG, 5 years of experience) [18]. This was done to make results from the two subgroups comparable, as the original prospective MR reading at UCLH was performed according to a 5-point Likert scale, but not PI-RADSv1.0. To detect potential differences or drawbacks of the RM using PI-RADSv1.0, all scans were retrospectively reported using PI-RADSv2.0.

### Biopsy protocol

Transperineal grid-directed biopsy performed under general anaesthesia is the standard technique at both sites, whose sPC-detection accuracy has been validated [17,19]. At Heidelberg, all men underwent transperineal fusion-targeted biopsy (FTB) with rigid (BiopSee, MedCom, Darmstadt, Germany) or elastic (Uronav, Invivo, Gainesville, USA) software-registration of MRI-suspicious lesions first (2–5 cores, median 2 per lesion) and then standard biopsy (SB) adjusted to PV (median 24 cores, according to the Ginsburg scheme) [17]. At UCLH, transperineal cognitive targeted biopsies were performed using the Hitachi Preirus (Hitachi Aloka Medical, Wallingford, USA) first, followed by SB using the Barzell 20-sector scheme [20]. Thus, both SB techniques were performed transperineally with an extended number of cores.

### Histopathology

Histopathological analyses were performed by or under supervision of uropathologists specialized in prostate assessment according to International Society of Urological Pathology standards. sPC was defined as Gleason score (GS)≥3+4 (WR, AS, AF).

### Assessment of the ERSPC-RC3

The ERSPC-RCs (www.prostatecancer-riskcalculator.com) are prediction models based on data of men in the ERSPC Rotterdam [21]. ERSPC-RC3 uses TRUS lesions, DRE, TRUS-measured PV and PSA. RC3 calculates the risk of finding any higher-grade (GS≥3+4) and/or locally advanced (T-stage≥T2c) PC in a conventional random-biopsy for men who have never been screened [21]. We retrospectively analysed ERSPC-RC3 on both subcohorts. ERSPC-RC3 were calculated manually per single patient using the original online RCs. These ERSPC-RCs were also used to combine ERSPC and PI-RADSv1.0 (ERSPC-R3+PI-RADSv1.0).

### Statistical analysis

Patient demographics, MRI and biopsy results were analysed descriptively, according to START recommendations (Table 1) [22]. Differences of the subcohorts of both sites were detailed (Table 2). For further information on how the RM was derived, see the original manuscript [10]. The regression equations were as follows:

$$\log\left(\frac{\pi_{i}}{1-\pi_{i}}\right)=-2.206+1.056\;\log\left(PSA_{i}\right)-0.021\;volume_{i}+0.018\;age_{i}+1.407\;I(DRE_{i}\geq cT2)-0.156\;I(PIRADS_{i}=2)+0.627\;I(PIRADS_{i}=3)+1.533\;I(PIRADS_{i}=4)+2.081\;I(PIRADS_{i}=5)$$

**Table 1:** Study population and results according to START criteria. Patients’ demographics including baseline clinical parameters, MRI and MRI/TRUS-fusion biopsy results according to START criteria.

Men included in analysis, n	293
Median Age, years (IQR)	65(58–70)
Median prebiopsy PSA-Level (IQR), ng/ml	7.2(5.0–11.5)
Suspicious DRE findings (≥T2), n (%)	104(35)
Median prostate volume (IQR), ml	42(30–60)
Median PSA density (IQR)	0.16(0.11–0.299
Men with PI-RADS≥3 lesions on mpMRI, n (%)	259(88)
Number of lesions PI-RADS≥3	319
Patients with one PI-RADS≥3 lesion	199
Patients with > 1 PI-RADS≥3 lesions	60
Overall PI-RADS score 3 lesions, n (% of PI-RADS≥3)	127(40)
Overall PI-RADS score 4 lesions, n (% of PI-RADS≥3)	102(32)
Overall PI-RADS score 5 lesions, n (% of PI-RADS≥3)	90(28)
Biopsies per patient, median (IQR)	27(24–30)
Systematic biopsies per patient, median (IQR)	23(19–26)
FTB per patient and per lesion, median (IQR)	4(2–6), 2(1–3)
Overall detection rate of prostate cancer, n (%)	176(60)
Men with significant prostate cancer, n (% of all men)	114(39)

n- Number, IQR- Interquartile range, PSA- Prostate specific antigen, ng- nanogram, ml- milliliter, DRE- Digital rectal examination, TRUS- transrectal ultrasound, mpMRI- multiparametric Magnetic Resonance Imaging, PI-RADS- Prostate Imaging Reporting and Data System, FTB- Fusion targeted biopsy

**Table 2:** Study population and results according to START criteria dichotomized into different cohorts. Differences in subgroups of UCLH and Heidelberg University Hospital for patient demographics, mpMRI PI-RADS scoring and biopsy results.

	Heidelberg cohort	UCLH cohort	*p*-value
No. of patients	160	133	
**Patient demographics**			
Median PSA level in ng/ml (IQR)	6.5 (4.9–11.0)	7.6 (5.3–13.0)	**0.042**
Median age, years (IQR)	65 (59–70)	64 (58–69)	0.391
Median prostate volume, ml (IQR)	40 (29–58)	45 (33–52)	**0.018**
Digital rectal examination (DRE), ≥cT2, n (% of all patients)	58 (36)	46 (35)	0.713
PSA-density (IQR)	0.18 (0.12–0.28)	0.18 (0.16–0.21)	0.947
**mpMRI PI-RADS score distribution (highest PI-RADS score per patient)**			0.154
No lesion/PI-RADS 1 (%)	13 (7)	1 (1)	
PI-RADS 2 (%)	9 (6)	11 (8)	
PI-RADS 3 (%)	50 (31)	41 (31)	
PI-RADS 4 (%)	44 (28)	38 (29)	
PI-RADS 5 (%)	44 (28)	42 (32)	
**Biopsy results**			
Median No. of cores (IQR)	28 (26–32)	26 (24–28)	0.995
Median No. of systematic cores (IQR)	24 (23–24)	24 (24–24)	0.889
Median No. of targeted cores (IQR)	4 (3–6)	2 (1–4)	0.614
No. of any prostate cancers (% of all men)	110 (69)	58 (44)	0.061
No. of significant prostate cancers (% of all men)	76 (48)	38 (29)	**0.019**

UCLH- University College London Hospital, IQR- Interquartile range, mpMRI- multiparametric Magnetic resonance imaging, SB- systematic biopsies, TB- targeted biopsies, PI-RADS- Prostate imaging reporting and data system, PC- prostate cancer, TRUS- transrectal ultrasound, PSA- prostate specific antigen

### Discrimination

**Fig 1:**
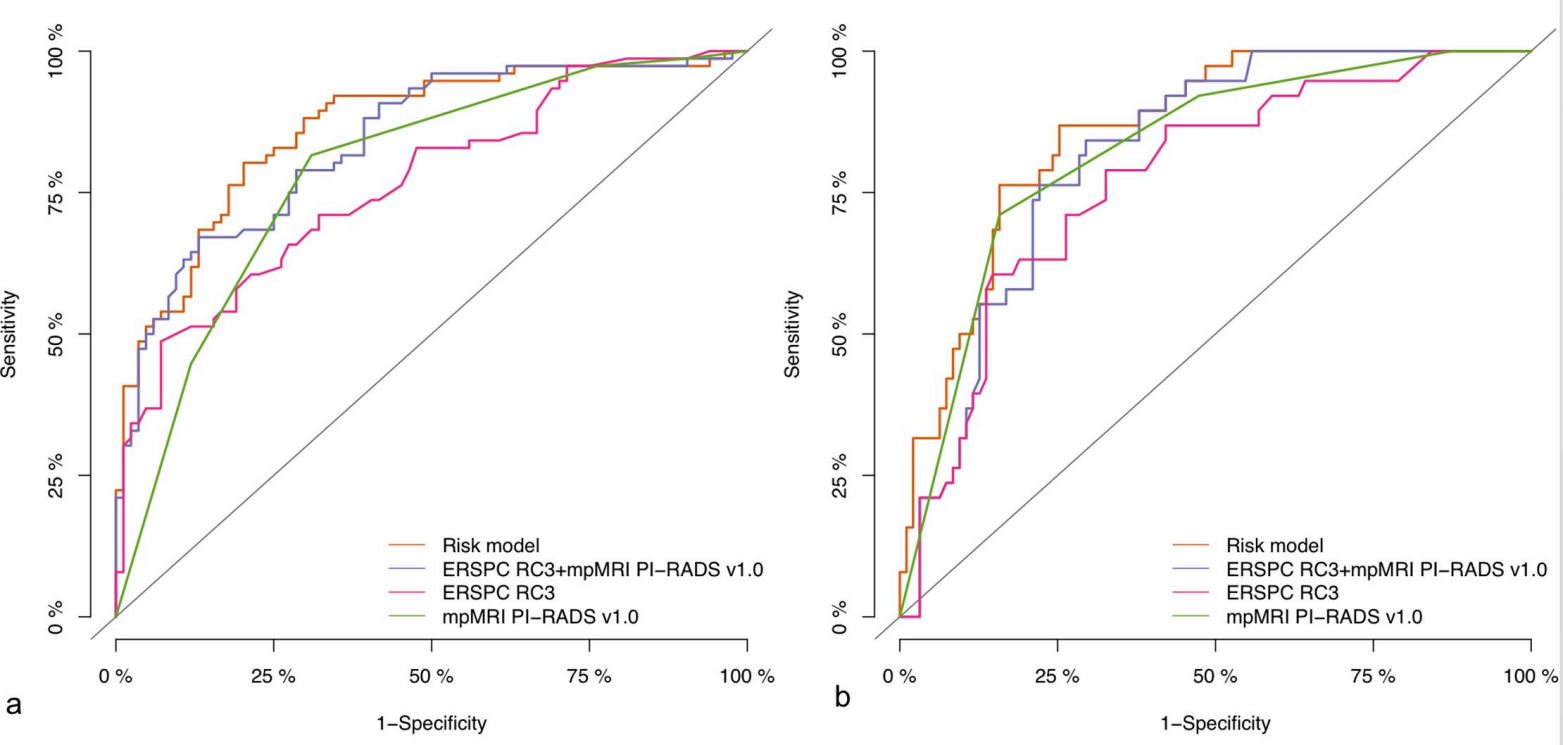
ROC curve analysis for the performance of mpMRI PI-RADSv1.0 (green line), ERSPC-RC3 (pink line), ERSPC-RC3+mpMRI PI-RADSv1.0 (purple line) and the risk model (orange line) to predict sPC for a) Heidelberg validation cohort, b) UCLH validation cohort. AUCs are given in Table 3.

Discrimination of ERSPC-RC3, PI-RADSv1.0, ERSPC-RC3+PI-RADSv1.0 and the RM were compared using Area-under-the-curve (AUC) of receiver-operating-characteristic (ROC) curve analysis (Fig 1). Statistical differences between predictive models were analysed using DeLong`s test and Holm adjustment for multiple testing (Table 3).

**Table 3:** Areas under the curve (AUC) of ROC curve analysis for the performance of mpMRI PI-RADSv1.0, ERSPC-RC3, the combination of ERSPC-RC3 and mpMRI PI-RADSv1.0 and the RM to predict sPC for a) Heidelberg validation cohort, and b) for men in the UCLH cohort.

Parameter	
a) Subset of biopsy-naïve men in the Heidelberg cohort (n = 160 available for RM validation)	AUC in ROC curve analysis (95% Confidence intervals)
Risk model	0.86 (0.81–0.92)
ERSPC RC3	0.77 (0.70–0.84)
ERSPC RC3 plus mpMRI PI-RADS v1.0	0.84 (0.78–0.90)
mpMRI PI-RADS v1.0	0.79 (0.72–0.85)
b) Subset of biopsy-naïve men in the UCLH cohort (n = 133 available for RM validation)	AUC in ROC curve analysis
Risk model	0.86 (0.80–0.92)
ERSPC RC3	0.77 (0.69–0.86)
ERSPC RC3 plus mpMRI PI-RADS v1.0	0.82 (0.75–0.89)
mpMRI PI-RADS v1.0	0.82 (0.75–0.90)
c) DeLong`s tests in the Heidelberg cohort after Holm adjustment for multiple testing	p-value
Risk model vs. ERSPC RC3	0.002
Risk model vs. ERSPC RC3 plus mpMRI PI-RADS v1.0	0.15
Risk model vs. mpMRI PI-RADS v1.0	0.005
d) DeLong`s tests in the UCLH cohort after Holm adjustment for multiple testing	AUC in ROC curve analysis
Risk model vs. ERSPC RC3	0.004
Risk model vs. ERSPC RC3 plus mpMRI PI-RADS v1.0	0.02
ERSPC RC3 plus mpMRI PI-RADS v1.0	0.2

ROC- Receiver Operating Characteristics, AUC- Area Under the Curve, ERSPC- European Randomised Study of Screening for Prostate Cancer, RC- Risk calculator, RM- Risk model, LR- Likelihood ratio, mpMRI- multiparametric Magnetic Resonance Imaging, PI-RADS- Prostate Imaging Reporting and Data System.

### Calibration of the RM

**Fig 2:**
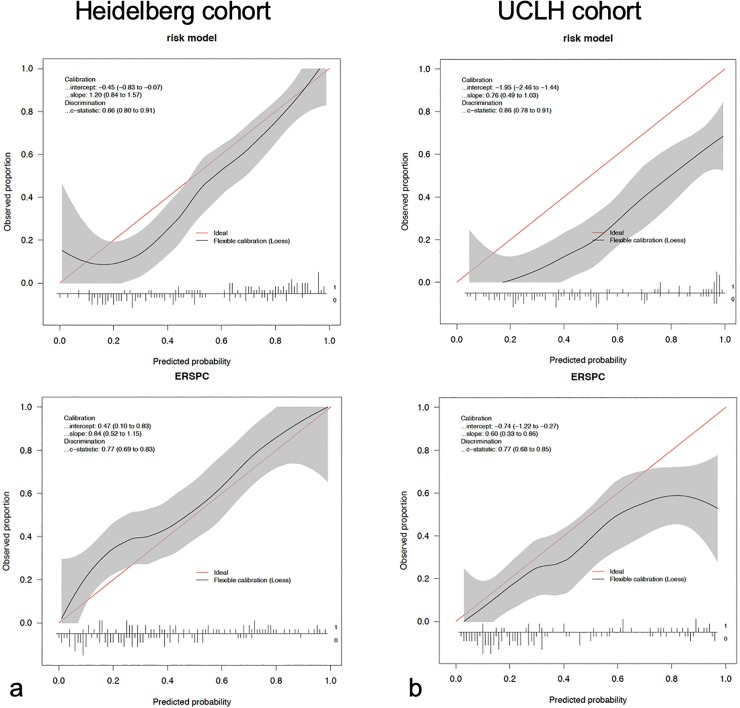
Calibration plots for the risk model and ERSPC-RC3 to predict sPC. a) Calibration plots for the Heidelberg validation cohort, b) Calibration plots for the UCLH validation cohort.

The extent of over- or underestimation of predicted probabilities relative to observed probabilities of sPC was explored graphically using calibration plots for both subcohorts. The calibration plot demonstrated that the RM overestimates event probabilities at UCLH (Fig 2). This seems to be mainly due to major differences between the prevalences of sPC in the Heidelberg training cohort and the UCLH validation cohort (46% and 29%, respectively). It is well known that one has to be cautious about extrapolating risk models built on populations very different from the population of interest [23]. Although the prevalence in the general population is comparable in Germany and the United Kingdom, the prevalence in academic centers might be variable. Therefore we thought to update our RM in terms of prevalence-adjustment and give both, the original and the adjusted risk model.

### Adjustment procedure for different prevalences

A modification of the nomogram to the present situation is to use the prior correction strategy described e.g. in Prentice and Pyke [24] and Janssen et al. [25].

Thereby, we correct the intercept of the RM by subtracting the log odds of the prevalence in the development cohort (0.456) and adding the log odds of the prevalence in the ERSPC-RC3 cohort (0.245) to provide equal calibration-in-the-large of the two prediction models:

logit (π_i) = -3.623 –logit(0.456) + logit(0.245) +0.550 log (PSA_i)-0.020 volume_i+ 0.034 age_i+0.712 I(DRE_i≥cT2)+0.299 I(PIRADS_i = 2)+1.198 I(PIRADS_i = 3)+1.841 I(PIRADS_i = 4)+ 3.094 I(PIRADS_i = 5)

with predictors PSA, prostate volume (in ml), DRE results, age (in years) and PI-RADSv1.0 [10].

However, note that this requires assumptions about the distribution of the population characteristics that might be critical. The resulting RM shifts the probabilities of sPC while keeping the scale fixed (the odds ratios of the predictors remain the same), thus only the last line of the nomogram is changed. This implies that discrimination performance is not affected.

### Decision curve analyses

**Fig 3:**
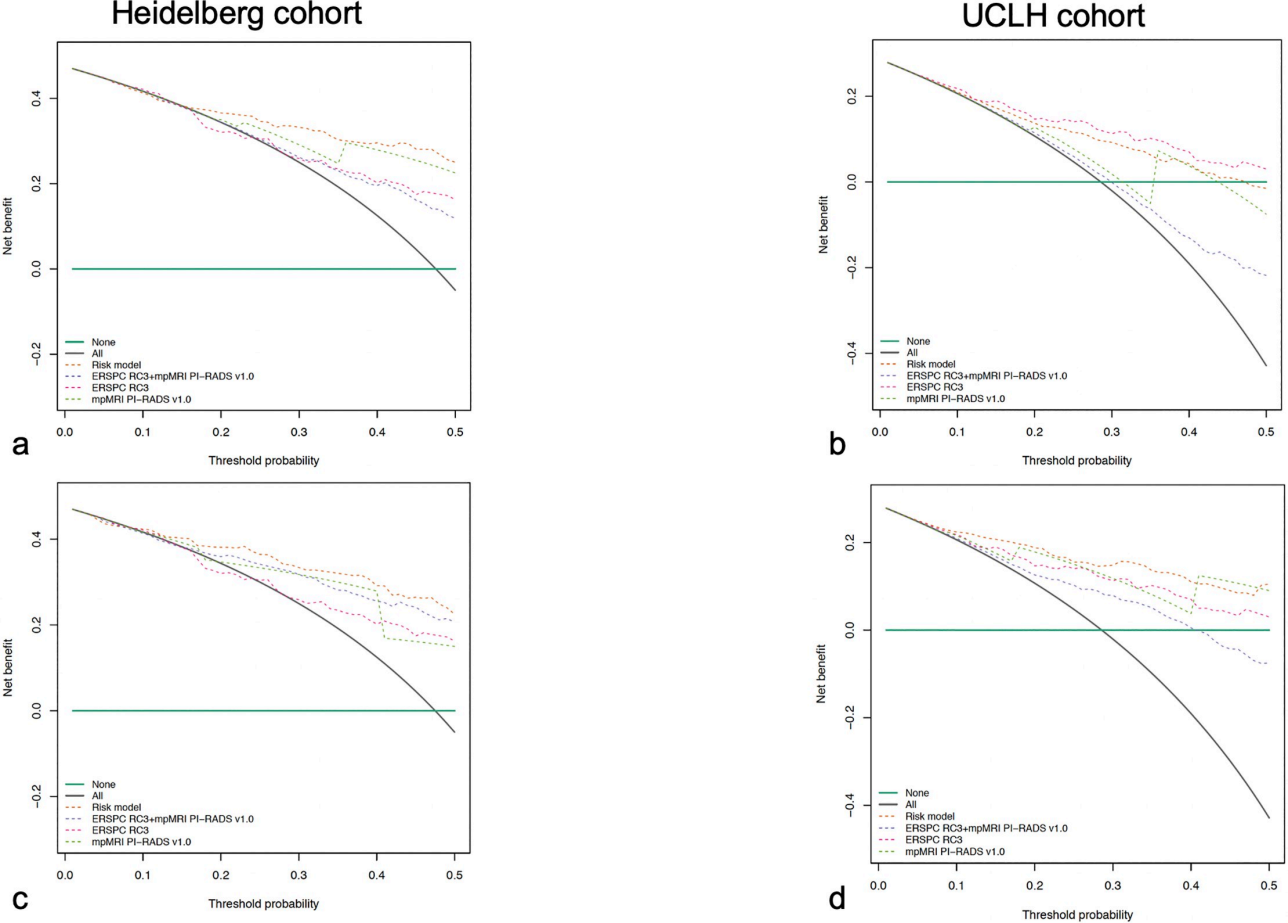
Net decision curve analyses demonstrating the benefit for predicting sPC on biopsy: a) for the unadjusted RM in the Heidelberg cohort, b) for the unadjusted model in the UCLH cohort, c) for the adjusted model in the Heidelberg cohort (according to the prevalence of the ERSPC 24.5%) and d) for the adjusted model in the UCLH cohort. The black line is the net benefit of providing all patients with MRI/TRUS-fusion biopsy and the horizontal green line is the net benefit of providing no patients with biopsy. The net benefit provided by each prediction tool is given (pink line for ERSPC-RC3, green line for mpMRI PI-RADSV1.0, purple for ERSPC RC3+mpMRI PI-RADSv1.0 and orange line for the RM).

Last, we assessed the clinical usefulness of the original and the prevalence-adjusted RM by using decision curve analysis (DCA)(Fig 3) [26]. We compared the RM and ERSPC regarding its clinical usefulness on DCA, performing a recalibration with respect to the ERSPC prevalence (24.5%), as mentioned before (Fig 3C and 3D).

Statistical analyses were performed using R version 3.5.0 (R Foundation for Statistical Computing, Vienna, Austria), packages ModelGood, DCA and rms [26]. Reporting followed Standards of Reporting of Diagnostic Accuracy (STARD) guidelines (S1 Table)[27].

## Results

In total 293 men underwent mpMRI and subsequent fusion-biopsy. Of them, 160 underwent procedures in Heidelberg und 133 men at UCLH. Patient demographics, MRI and biopsy data are given in Table 1. sPC was detected in total 114 patients (39%). Stratification into centers revealed sPC in 76 men (48%) in the Heidelberg and 38 (29%) men in the UCLH cohort.

Differences on patients’ demographics, mpMRI and biopsy outcomes between the both subcohorts are given in Table 2.

The discrimination of the RM was compared to ERSPC-RC3, PI-RADSv1.0 and ERSPC-RC3+PI-RADSv1.0 using ROC curve analyses (Fig 1, Table 3). In the Heidelberg validation cohort, the RM reached a higher AUC (0.86), compared to ERSPC-RC3 (0.77), and PI-RADSv1.0 (0.79; section a of Fig 1, section a of Table 3). The RMs AUC was comparable to ERSPC-RC3+PI-RADSv1.0 (0.84). At UCLH, the discrimination of the RM (0.86) was superior to ERSPC-RC3 (0.77) and comparable to PI-RADSv1.0 (0.82) and ERSPC-RC3+PI-RADSv1.0 (0.82)(Table 3).

Calibration plots of the RM (Fig 2A and 2B) demonstrate that there are no untoward deviations of predicted from observed risk of sPC over the entire range in the Heidelberg subgroup. At UCLH, the calibration plot demonstrated systematic overestimation of the sPC probability for both, the RM as well as ERSPC-RC3 (Fig 2B), but for ERSPC-RC3 to a much smaller extend.

In adjusted (with respect to the original ERSPC prevalence of 24.5% of sPC) DCA the RM had a higher net benefit in terms of accurately detecting patients with sPC compared to mpMRI and the ERSPC-RC (Fig 3C and 3D) [21]. The RM showed a benefit for sPC threshold probabilities above 10% at both centres (Fig 3C and 3D).

## Discussion

The results of our study showed that the novel RM, combining clinical and MRI parameters discriminates well in both, a consecutive validation cohort of the same institution on whose patients the RM was developed, and an external cohort. The discrimination performance of the RM was similar in both cohorts and significantly better than the performance of ERSPC-RC3. This is in line with recent results demonstrating added benefit of MRI in combination with clinical parameters [11,15].

Second, this study shows that the present RM performs well in two different cohorts using different MRI scanners and radiologists with different experience in prostate-MRI. Our results furtherly support that standardized mpMRI reading is reliable not only within a nomogram combining MRI and clinical parameters, but also as a standalone screening test. The discrimination performance of expert MRI reading was comparable, with an AUC of 0.82 at UCLH and 0.79 in Heidelberg. However, DeLong`s test demonstrated that adding clinical parameters within the RM significantly enhances the accuracy of MRI alone to predict sPC in Heidelberg, but not at UCLH.

A third point is the comparison to the clinical ERSPC-RC. Additional benefit of adding MRI to ERSPC-RC3 has recently been shown in a multicentric study, with comparable results [15]. Even if the novel RM outperforms the ERSPC-RC alone, our study could demonstrate that the ERSPC-RC3 alone has a good discrimination performance. Considering that Poyet et al. recently demonstrated that the performance of a nomogram depends on the number of biopsy cores and decreases with the increase of the cores taken, we could support the generalizability of the ERSPC-RC3, which was externally validated with an accurate AUC of 0.77 in both cohorts using extended biopsy protocols [21,28].

Additionally, in the light of the results of Poyet et al., who could demonstrate that an increased number of cores taken (12 core compared to 6–8 core) negatively influenced the performance of a validated risk model, the use of a robust reference test, combining extended SB and FTB with a median cores of 27, as in the present study, emphasizes the discrimination performance of the novel RM in the ROC curve analyses [28]. However, regarding the clinical applicability of the RM, we have to acknowledge that the RM systematically overestimated the sPC probability in a subgroup with lower prevalence (UCLH subgroup) than in the cohort on which the RM was developed (Heidelberg subgroup). Thus, the clinical applicability of the RM in the external validation needs to be discussed. In particular, this study emphasizes the prevalence-dependence of risk models.

To analyse the real net benefit of a novel RM, it has to be adjusted according to the actual prevalence of the considered population. This is critical, because one has to assume the prevalence of the cohort, as the prevalence is unknown in most cases. This is important, if one wants to extrapolate the RM and consecutively to introduce it in common clinical practice.

However, this is a general problem in any risk model. We acknowledge that any risk model is only applicable on comparable populations and extrapolation to populations with different characteristics is problematic. Alternatively, when different prevalences occur, the intercept has to be adjusted for correct risk assessment. However, we emphasized that this does not affect discrimination performance in external validation. Moreover this is a reflection to a general problem of risk modeling.

Furthermore, in clinical practice, both physicians and different patients have different trade-offs of individuals`sPC risk. Therefore there are no single risk thresholds and DCA is the most informative tool to weigh the relative harm of potentially unnecessary biopsy and the benefit of diagnosing sPC.

In our study, the discrimination performance of the original RM, analyzed by ROC curve analyses (Fig 1, Table 3) was good in both, a cohort that is comparable in terms of prevalence to the cohort in which the model has been derived (Heidelberg), and also in a cohort, in which the prevalence is different (UCLH). It is well known that ROC curve analyses are not affected by prevalences. However, individuals`predicted probabilities of sPC are trivially affected by the prevalences. The predicted and the empirical sPC probability was visualized in calibration curves, demonstrating that the original model overestimated the probabilities due to prevalence differences (Fig 2B). In decision curve analyses the net benefit of the original model was therefore limited in the cohort in which the prevalence is smaller, because the patients`individual probability is overestimated (Fig 3B). After adjustment of the RM such that prevalences underlying the RM and ERSPC-RC are equivalent has been performed, as in the Material and methods section, the overestimation is extenuated (compare Fig 2B, in which the upper curve gives the calibration of the original model as compared to the adjusted one in the lower curve). This also affects the decision curve analysis, demonstrating improved benefit of the adjusted RM over a threshold probability from 0.10 to approximately 0.50 (Fig 3D).

Our study has limitations. First, as mentioned before, prevalence-dependence of the RM limits its generalizability. In order to correctly determine the individuals`risk of harbouring sPC, it is mandatory to be aware of the sPC prevalence in the current population or to assume this to adjust the RMs`intercept.

Both cohorts used a similar biopsy approach including MRI/TRUS-fusion and template SB with an extended number of biopsy cores. Therefore, we cannot clearly state how the RM performs on cohorts using different biopsy strategies (e.g. using widespread standard 12-core TRUS-biopsy). However, the application of a robust reference test is a strength of the study, and the relative poor performance of the 12-core TRUS-biopsy has been recently confirmed [3,4]. One might assume that differences occur due to different fusion-biopsy techniques. With regards to the biopsy approach the available data in the literature is rather scarce. The latest review and clinical trials were not able to show significant differences between cognitive and software-assisted biopsy for MRI-targeted cores, but one of the limitations given were limited amounts of studies or patients, respectively [29–32]. Additionally, Wysock et al. compared MRI/TRUS-fusion-guided biopsies versus visual estimated targeting in a prospective study including 125 men with suspicious lesions [33]. They found that MRI/TRUS-fusion-guided biopsies had a slightly improved cancer detection rate compared to visual estimation for GS≥3+4 (20% versus 15%, p = 0.05). Puech et al. observed no difference in the cancer detection rate of PC for rigid software co-registration using software-guided compared to cognitive TB (53% versus 47%)[34]. However, Delongchamps et al. reported that cognitive fusion-biopsy was not significantly better than systematic biopsies, while both software co-registration devices tested (Esaote/MyLabTMTwice and Koelis/Urostation) significantly increased the cancer detection rate compared to systematic biopsies using logistic regression analysis in a cohort of 391 patients. In our study, the additional systematic biopsy part of the biopsy procedure was comparable in both centers, using a transperineal extended SB scheme with a median of 24 cores. In a subanalysis (data not shown) we could demonstrate that in the Heidelberg subgroup the software-assisted approach missed 4% of sPC as compared to the systematic saturation biopsy, whereas the cognitive-fusion biopsy approach at UCLH missed 9% of sPC. Therefore, the differences in prevalence are not fully explainable by different TB approaches.

We prospectively used PI-RADSv1.0, while v2.0 has recently become the favoured approach, since the time period for data accrual predominantly was before publication of v2.0. As the original RM was constructed using v1.0, the retrospective MR reading at UCLH also used v1.0. However, data are still not fully decisive as to the advantage of either over the other version. In a recent meta-analysis, a higher pooled sensitivity (0.95) for v2.0 compared to v1.0 (0.88) but a comparable pooled specificity (0.73 versus 0.75) were detected [35]. Contrary, Auer found a significantly larger discrimination for PI-RADSv1.0 (0.96) versus v2.0 (0.90) [36]. However, utilization of PI-RADSv1.0 is a common problem of recent RMs, and data on the impact of v1.0 versus v2.0 on RMs are lacking [10,15]. When retrospectively reading MR scans according to PI-RADSv2.0, we could demonstrate the discrimination performance of PI-RADSv2.0 in the UCLH cohort was higher than for PI-RADSv1.0 (AUC 0.89), but slightly lower in the Heidelberg subgroup (AUC 0.75). In addition, the RMs performance at UCLH would be improved when replacing PI-RADSv1.0 by v2.0 (AUC 0.89). Results for ROC curve analyses and Decision curve analyses are given in S2 Fig. However, we did not develop a completely novel RM, as the purpose of our study was to validate an existing one. Additionally, when all available risk models combining clinical and MR parameters are analysed, the predictive performance of standardized MR reading is comparable when using PI-RADSv1.0 in the studies of Alberts et al., van Leeuwen et al. and the original RM, or PI-RADSv2.0 in the study of Mehralivand et al. [10,11,15,37]. All risk models enhance the discrimination compared to MRI scoring and clinical parameters alone. Further investigations are needed to investigate the performance of a novel RM including PI-RADSv2.0.

In addition, costs and capacity of MRI as an upfront test have to be considered. However, the cost-effectiveness of using upfront mpMRI has been demonstrated recently [38].

The sample size of this study population is limited, in particular in the UCLH cohort. This might also affect the applicability of both, the RM and the study`s results. However, by analysing subcohorts of consecutive men in both centers, potential bias by patient selection should be decreased.

Lastly, our sPC definition of GS≥3+4 is debatable. However, to compare our results with ERSPC-RC3, GS≥3+4 was appropriate. We acknowledge that the comparison to ERSPC-RCs is not perfect, since our sPC definition did not include clinical T-staging [21]. Additionally, tumor involved core length / cancer core length had not available. Thus we could not include it into the definition of sPC.

The conclusion of this study is that the novel RM can improve the prediction of harboring sPC. If prevalence is properly acknowledged, the RM provides benefit in the decision-making process of which patient should be biopsied over clinical risk modeling and imaging alone.

## Supporting information

S1 Table Standards of Reporting of Diagnostic Accuracy (STARD) checklist. (DOCX)

S2 Table Raw data of study population. (CSV)

S1 Fig Study flowchart with inclusion criteria. (JPG)

S2 Fig ROC curve analysis (a and b) and Net decision curve analysis (c and d) for the performance of mpMRI PI-RADSv1.0 (green line), ERSPC-RC3 (pink line), ERSPC-RC3+mpMRI PI-RADSv1.0 (purple line), the risk model (orange line), mpMRI PI-RADSv2.0 (grey line), ERSPC-RC3+mpMRI PI-RADSv2.0 (brown line) and the risk model with mpMRI PI-RADSv2.0 (yellow line) for Heidelberg validation cohort and UCLH validation cohort. On Net decision curve analysis, the black line is the net benefit of providing all patients with MRI/TRUS-fusion biopsy and the horizontal green line is the net benefit of providing no patients with biopsy. The net benefit provided by each prediction tool is given.
(PNG)

## Abbreviations

AUC: Area-under-the-curve
DCA: Decision curve analysis
DCE: Dynamic contrast-enhanced Imaging
DRE: Digital-rectal examination
ERSPC: European Randomised study of Screening for PC
FTB: MRI-fusion targeted biopsy
GS: Gleason score
log: logarithmic
mpMRI: multiparametric Magnetic Resonance Imaging
MRI: Magnetic Resonance Imaging
PC: Prostate cancer
PI-RADS: Prostate Imaging—Reporting and Data System
PSA: Prostate specific antigen
PV: Prostate volume
RC: Risk calculator
RM: Risk model
ROC: Receiver-operating-characteristics
RP: Radical prostatectomy
SB: Systematic transperineal saturation biopsy
sPC: Significant prostate cancer
STARD: Standards of Reporting of Diagnostic Accuracy
START: Standards of Reporting for MRI-targeted Biopsy Studies
TRUS: transrectal ultrasound
TV: Tumor volume
UCLH: University College London Hospital
