# Supplementary figures and images for: Prediction of significant prostate cancer in biopsy-naïve men: Validation of a novel risk model combining MRI and clinical parameters and comparison to an ERSPC risk calculator and PI-RADS
Source: PLoS One. 2019 Aug 26;14(8):e0221350. doi: 10.1371/journal.pone.0221350 (PMC6710031; doi:10.1371/journal.pone.0221350)

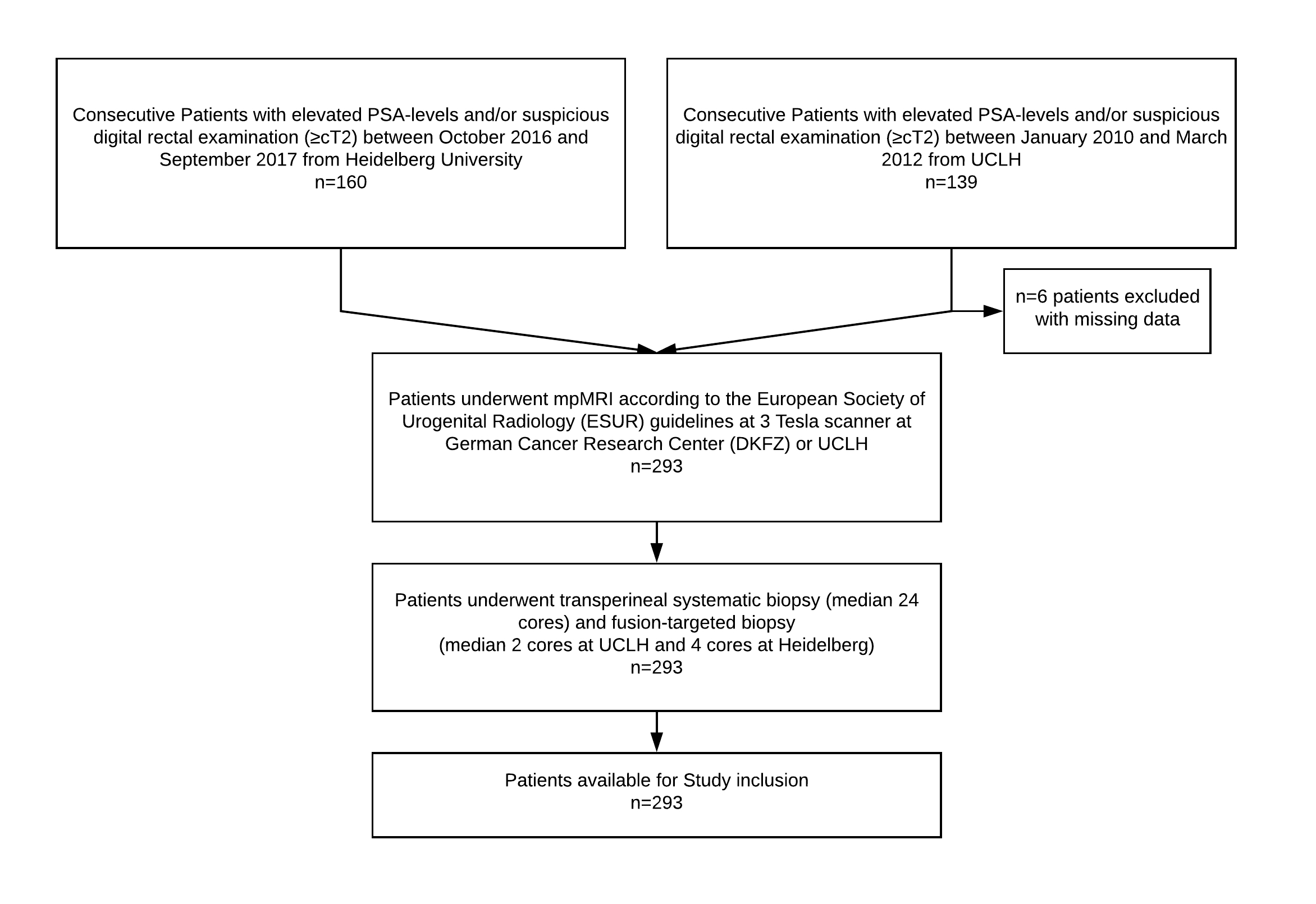

Supplement: S1 Fig — (JPG) [file pone.0221350.s003.jpg]

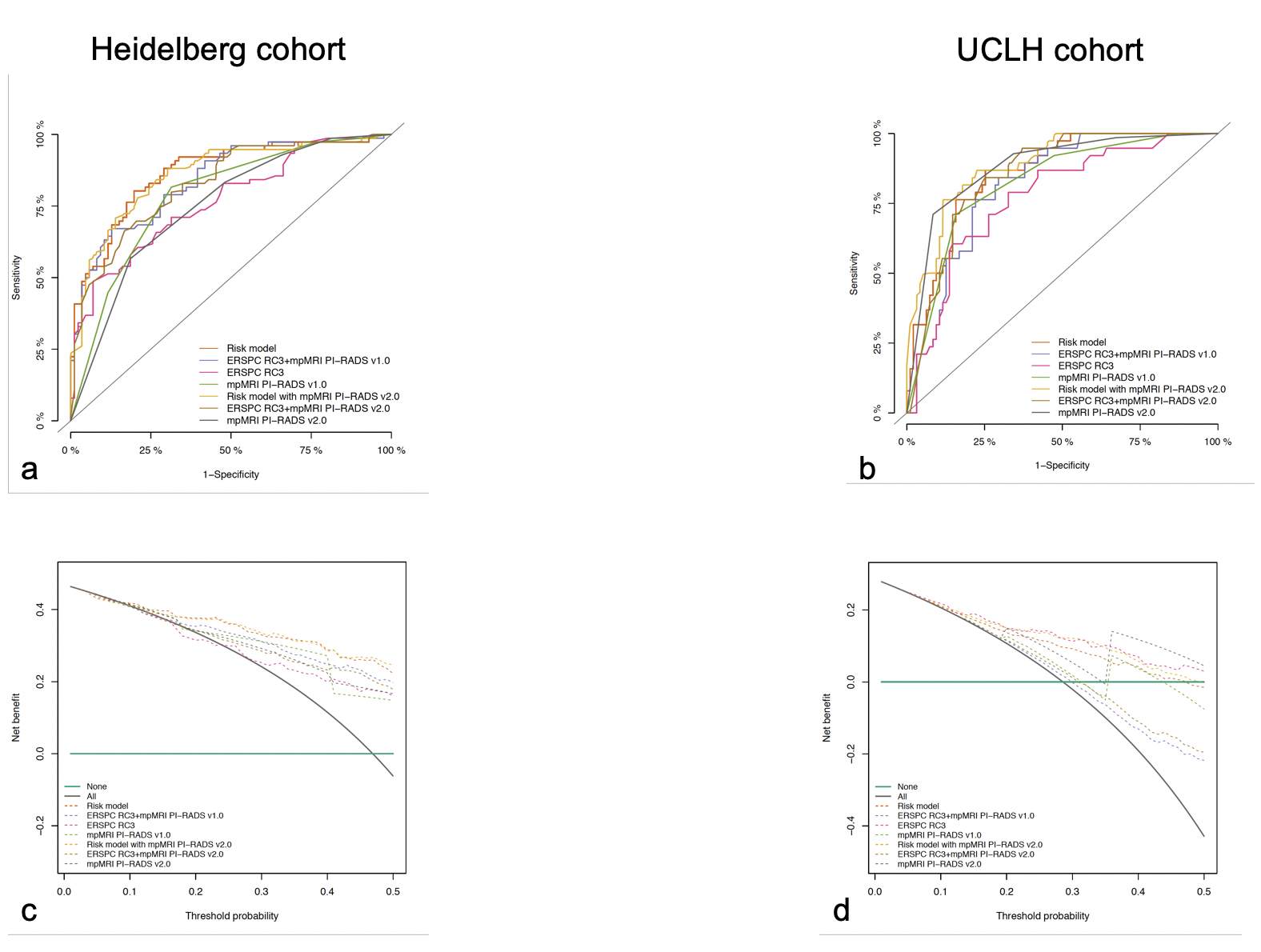

Supplement: S2 Fig — ROC curve analysis (a and b) and Net decision curve analysis (c and d) for the performance of mpMRI PI-RADSv1.0 (green line), ERSPC-RC3 (pink line), ERSPC-RC3+mpMRI PI-RADSv1.0 (purple line), the risk model (orange line), mpMRI PI-RADSv2.0 (grey line), ERSPC-RC3+mpMRI PI-RADSv2.0 (brown line) and the risk model with mpMRI PI-RADSv2.0 (yellow line) for Heidelberg validation cohort and UCLH validation cohort. On Net decision curve analysis, the black line is the net benefit of providing all patients with MRI/TRUS-fusion biopsy and the horizontal green line is the net benefit of providing no patients with biopsy. The net benefit provided by each prediction tool is given. (PNG) [file pone.0221350.s004.png]
